# Self-assembly of silicon nanowires studied by advanced transmission electron microscopy

**DOI:** 10.3762/bjnano.8.47

**Published:** 2017-02-15

**Authors:** Marta Agati, Guillaume Amiard, Vincent Le Borgne, Paola Castrucci, Richard Dolbec, Maurizio De Crescenzi, My Alì El Khakani, Simona Boninelli

**Affiliations:** 1 Dipartimento di Fisica e Astronomia, Università di Catania, Via S. Sofia 64, Catania 95123, Italy; 2 CNR IMM-MATIS, Via S. Sofia 64, Catania 95123, Italy; 3 Institut national de la recherche scientifique, Centre-Énergie, Matériaux et Télécommunications (INRS-EMT), 1650 Blvd. Lionel Boulet, Varennes QC-J3X 1S2, Canada; 4 Institut Pprime, UPR 3346, CNRS - Université de Poitiers, ISAE-ENSMA, 11 Boulevard Marie et Pierre Curie, 86962 Futuroscope-Chasseneuil, France; 5 Dipartimento di Fisica, Università di Roma “Tor Vergata”, Via della Ricerca Scientifica 1, Roma 00133, Italy,; 6 Tekna Plasma Systems Inc., 2935 Industrial Blvd., Sherbrooke QC-J1L 2T9, Canada

**Keywords:** silicon nanowires, transmission electron microscopy, vapor–liquid–solid growth

## Abstract

Scanning transmission electron microscopy (STEM) was successfully applied to the analysis of silicon nanowires (SiNWs) that were self-assembled during an inductively coupled plasma (ICP) process. The ICP-synthesized SiNWs were found to present a Si–SiO_2_ core–shell structure and length varying from ≈100 nm to 2–3 μm. The shorter SiNWs (maximum length ≈300 nm) were generally found to possess a nanoparticle at their tip. STEM energy dispersive X-ray (EDX) spectroscopy combined with electron tomography performed on these nanostructures revealed that they contain iron, clearly demonstrating that the short ICP-synthesized SiNWs grew via an iron-catalyzed vapor–liquid–solid (VLS) mechanism within the plasma reactor. Both the STEM tomography and STEM-EDX analysis contributed to gain further insight into the self-assembly process. In the long-term, this approach might be used to optimize the synthesis of VLS-grown SiNWs via ICP as a competitive technique to the well-established bottom-up approaches used for the production of thin SiNWs.

## Introduction

As the scaling down of the feature size of devices proceeds [1], new synthesis routes are being explored to produce materials with ultra-low dimensionality to be used as building blocks to improve the functionality of next-generation devices [2]. So far, conventional manufacturing processes based on top-down methods have been employed in Si-based microelectronics. These methods have encountered, among others, a non-trivial issue related to the cost of the sequential steps required to achieve the desired nanostructure and to the scaling up of such procedures. On the other hand, bottom-up approaches, founded on the aggregation of atoms or molecules as elementary components for the synthesis of nanomaterials, seem to be a good strategy to fabricate ultra-small structures. This concept encompasses the physics and the chemistry of nanostructure formation via a “self-assembly” route. Such ultra-low dimensional systems require appropriate characterization tools, which may lead to further insight in the comprehension of the dynamics of nanostructure formation. Transmission electron microscopy (TEM) has been the principal imaging and analytical technique for the characterization of materials at the nanoscale. Quite recently, subangstrom resolution has been reached in scanning TEM (STEM) mode, thanks also to the improvements in aberration correctors [3]. In order to access a more realistic three-dimensional vision of nanomaterial components, 3D characterization techniques are highly demanded. For this purpose, atom probe tomography (APT) [4] and X-ray tomography [5] have been used. Nevertheless, while X-ray tomography has a rather limited spatial resolution (≈2 µm), APT offers better resolution (up to the single atom detection) but has the disadvantages that a limited volume can be measured (no more than 100 nm^3^) and the sample is destroyed during analysis. In this context, electron tomography (ET) together with TEM arises as a remarkable technique to study a larger range of volumes, while still offering reasonable spatial resolution from ≈1 nm^3^ [6] down to atomic resolution in very recently developed microscopes [7–8]. Electron tomography is accomplished through the reconstruction of a sequence of projection images acquired by tilting the TEM sample holder. However, to achieve an accurate 3D reconstruction, all the images of the series should obey the “projection requirement”, which states that the intensity of each micrograph must be a monotonic function of the physical property of the object [9]. It is well known that conventional bright field (BF) and dark field (DF) imaging are dominated by diffraction contrast. Thus in crystalline samples, the contrast changes abruptly as long as the beam axis intercepts the different crystalline zone axes. More recently, this issue has been overcome whereas the improvement of high angular annular dark field (HAADF) in STEM associated with ET has been confirmed as the most appropriate mode to image crystals, since it meets the projection requirement, associating the contrast to the atomic number [6]. By combining the HAADF and ET techniques along with energy dispersive X-ray (EDX) spectroscopy, it is possible to gather both imaging and analytical information at the same time.

In the present work, ET combined with STEM-EDX and energy-filtered TEM (EFTEM) enabled the structural characteristics of SiNWs spontaneously assembled during an inductively coupled plasma (ICP) process to be elucidated as finely as possible. The ICP technique has been conventionally exploited for the synthesis of micrometer-structured Si spheres [10]. In this regard, the formation of one-dimensional nanostructures such as SiNWs within the micrometer-sized spherical particles should be explained. In particular, we focus our study on ICP-produced SiNWs (less than 5% of the whole population) on which a peculiarity exists in the form of a high-contrast nanoparticle at the top. However, conventional 2D TEM imaging would not be able to definitively demonstrate whether the nanoparticle is embedded inside the SiNW or located at the tip. To unequivocally resolve this point, 3D STEM tomography characterization at the nanoscale was employed. Finally, the metallic composition of such nanoparticles was ascertained via the synergetic use of EFTEM and STEM-EDX. The ensemble of our results suggests that the vapor–liquid–solid (VLS) mechanism is the driving process for the growth of these SiNWs and could open the route for the production of SiNWs via the ICP technique.

## Results and Discussion

**Figure 1:**
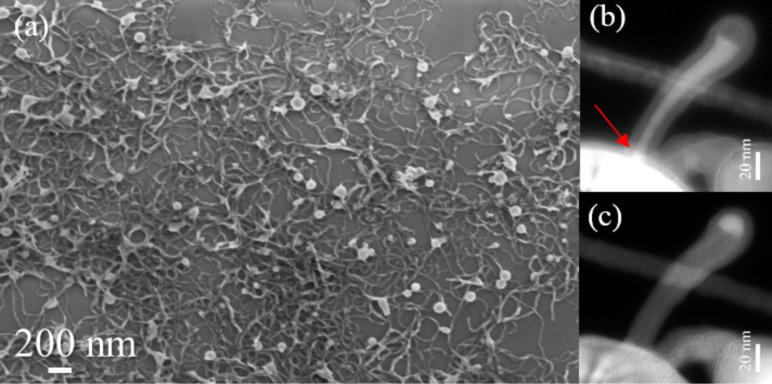
(a) Typical SEM image showing the morphology of the as-collected sample; EFTEM images obtained at (b) the Si plasmon loss (17 eV) and (c) the SiO_2_ plasmon loss (23 eV), revealing the Si–SiO_2_ core–shell structure and the structural continuity between the Si core of the SiNW and the SiNS, as indicated by the red arrow in (b).

Preliminary examinations of the ICP sample were performed by means of scanning electron microscopy (SEM). In the typical SEM image, as reported in Figure 1a, the presence of both nanospheres (NSs) and nanowires (NWs) can be observed. Statistical analyses conducted on hundreds of nanostructures allowed us to estimate that the diameter of the NSs range from 50 to 500 nm, while the NW length varies from ≈100 nm up to ≈2–3 μm. Energy-filtered TEM (EFTEM) images, acquired in correspondence to the Si plasmon loss (17 eV) and SiO_2_ plasmon loss (23 eV), display a common core–shell Si–SiO_2_ internal structure for both the NSs and the NWs (see Figure 1b and Figure 1c). Further EFTEM investigations conducted on hundreds of NWs revealed that their structural characteristics, in terms of length and diameter distribution, can be associated with two main families of SiNWs: longer SiNWs (2–3 μm) exhibiting an ultra-thin diameter (2–3 nm) and shorter SiNWs (maximum length of ≈300 nm) with a diameter of a few tens of nanometers. In this paper we focus our study on NWs belonging to the second family. In fact, the reason for our investigation stems from a peculiarity of these SiNWs, namely the presence of a high-contrast nanoparticle on the top (diameter ≈15 nm) corresponding to the SiNW core diameter, as shown in the EFTEM images in Figure 1b and Figure 1c.

**Figure 2:**
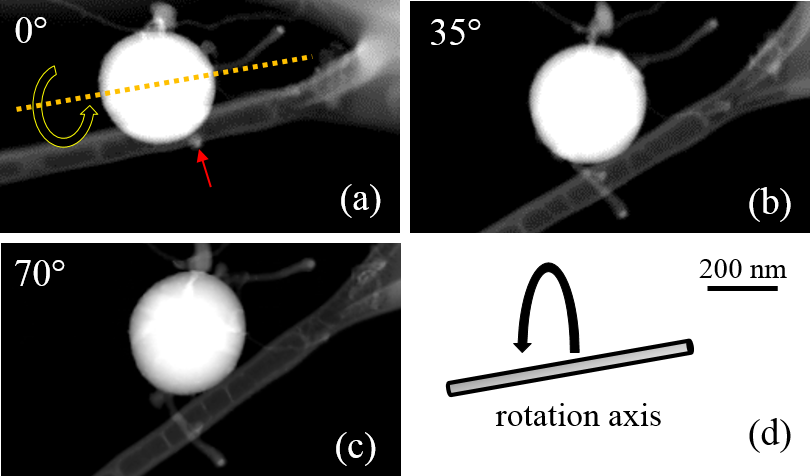
HAADF-STEM micrographs of a SiNS with two connected SiNWs, acquired at (a) 0°, (b) 35° and (c) 70° with respect to the rotation axis shown in (d), which corresponds to the axis of one of the two SiNWs, as indicated by the yellow dashed line in (a). The other SiNW, indicated by the red arrow in (a), is not visible at a rotation angle of 0°, but becomes evident after rotation of the sample (b and c).

Electron tomography and STEM-EDX analyses were conducted on these short SiNWs in order to better elucidate their structural and chemical characteristics. 3D tomography was performed on the nanostructures shown in Figure 2a–c, where three HAADF-STEM images of a SiNS with two SiNWs, acquired at tilting angles of 0°, 35° and 70°, are reported. The axis of rotation of the tomography measurement sample holder, depicted in Figure 2d and superimposed on the HAADF-STEM image in Figure 2a for a better understanding, was nearly aligned to the top right NW in Figure 2a. This allowed us to tilt the sample from −50° to +57° with a step of 2°, without any shadowing effect caused by the C networked structure of the TEM grid. Image shift compensation and focus were manually adjusted during acquisition. With this procedure, we acquired 55 images at different projections in about two hours.

**Figure 3:**
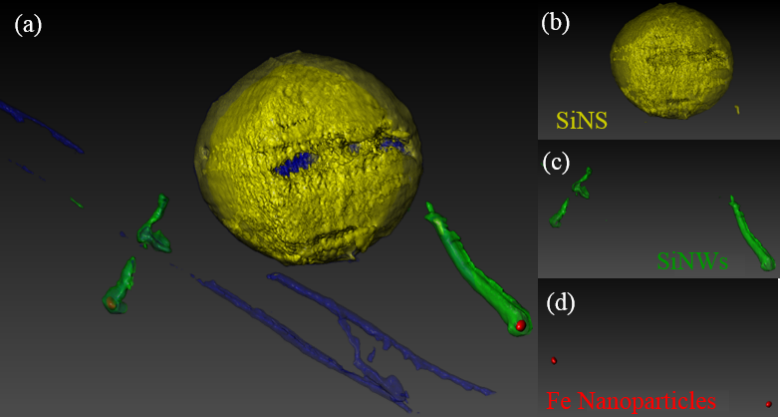
(a) Volume reconstruction of the system formed of (b) a SiNS and (c) two SiNWs having a Fe nanoparticle at the top, as illustrated in (d).

Nevertheless, from the inspection of Figure 2a alone, it is not possible to discern the nature of another Si nanostructure, indicated by the red arrow in Figure 2a. The presence of a second SiNW appears when the nanostructure is tilted at 35° with respect to the rotation axis, which is better shown in Figure 2b. Moreover, the inspection of the projection at 70° cleary demonstrates that both SiNWs exhibit a nanoparticle on top. The chemical composition of this nanoparticle was investigated by STEM-EDX and will be discussed hereafter. The reconstructed 3D structure, comprised of the SiNWs connected with the SiNS, is represented in Figure 3. Additionally, a video showing the 3D reconstructed volume, from which the corresponding image in Figure 3 was extracted, is reported in Supporting Information File 1. The shape and the location of the nanoparticles can be clearly identified. In fact, the different elements of the reconstructed volume have been shown separately in Figure 3b–d.

The identification of these elements was accomplished by performing the same reconstruction four times and separating the four different areas of interest. Thus, it is possible to distinguish the SiNS in yellow, the two SiNWs in green, the two nanoparticles in darkish red and the carbon support layer in blue in Figure 3. From this 3D reconstruction, we can conclude that the nanoparticles are located on the top of the SiNWs, and are not embedded inside of them, as it could be misleadingly inferred by a conventional 2D TEM image. On the other hand, due to the poor contrast at the base of the SiNWs, it was not possible to reconstruct the SiNW–SiNS interface. However, this issue is clarified by the EFTEM images, as depicted in Figure 1b, which demonstrate the structural continuity between the SiNW core and the SiNS (indicated by the arrow). This indicates the Si substrate on which the NWs grow before further oxidation occurs, which is induced by the oxygen present in the ICP chamber. Indeed, the oxygen derives from the native oxide of the Si powder feedstock, which is released during the spheroidization process in the ICP reactor.

**Figure 4:**
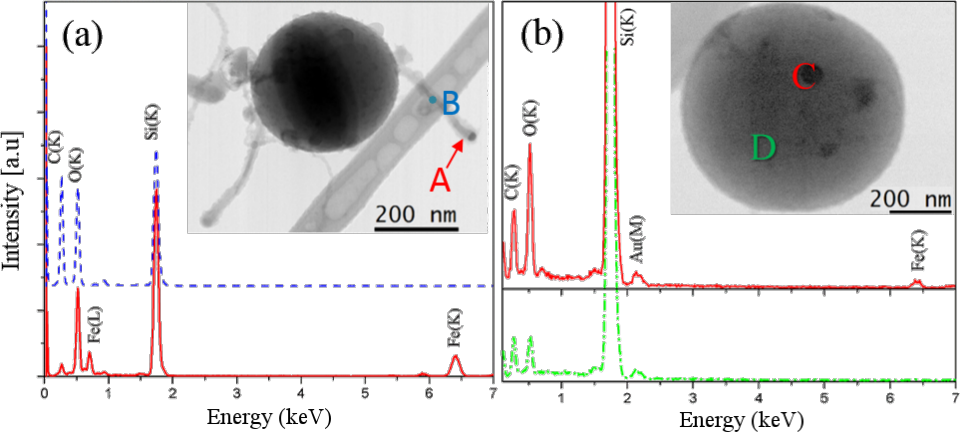
STEM-EDX spectra acquired at the points indicated in the BF STEM image in the insets: (a) SiNWs growing from the underling SiNS and (b) SiNS with Fe nanoparticles on the surface.

Statistical STEM-EDX analyses were carried out using a sub-nanometer electron probe in order to corroborate the structural analysis with further chemical information. Typical EDX spectra were acquired in two different regions over the SiNWs, namely (i) at the dark particle (indicated by point A in the inset in Figure 4a), and (ii) along the SiNW (indicated by point B in Figure 4a). The spectra, reported in Figure 4a, clearly evidenced that only Si and O peaks are present along the SiNW, which is in agreement with the previous EFTEM analyses. The C peak comes from the underlying lacey carbon Cu TEM grid used as a supporting substrate for the analysis, representing a background for our study. More interestingly, the presence of Fe was detected in correspondence with the nanoparticle at the tip of the SiNW, while no iron signal was found along the SiNW, within the sensitivity of our EDX measurements (i.e., less than 1 atom %).

Calculations based on the ZAF method [11] applied to our STEM-EDX analyses revealed that the nanoparticle at the tip was comprised of 7 atom % Fe and 14 atom % Si. Thus, we can infer that the nanoparticles are composed of a FeSi_2_ alloy. This result is in agreement with that previously reported in [12], where we demonstrated using high-resolution TEM imaging that the interplanar distance in the nanoparticle is compatible with a FeSi_2_ alloy. Such a composition corresponds to the most stable Fe–Si-based alloy at high temperature (≈10,000 K) under conditions of an abundance of Si content, as established by the Fe–Si binary phase diagram [13].

It is well known that metal-containing particles located at the top of SiNWs are a distinctive feature of VLS-grown SiNWs, which occurs on crystalline Si substrates [14–15]. Hence, we argue that the growth of short SiNWs via the above-described ICP process occurs through the VLS mechanism catalyzed by iron nanoparticles. The origin of the nanoparticles is the impurities present in the initial Si powder feedstock (containing 0.18 atom % Fe impurities), while the Si core of the NSs acted as the local substrate where the growth occurs. Indeed, the Fe nanoparticles are expected to form at the outer surface of the larger SiNSs formed during the spheroidization process. Once the Fe nanoparticles are present at the SiNS surface, the SiNW synthesis can occur via supersaturation of the Si vapor in the catalytic nanoparticle and precipitation of Si, which then crystallizes in the form of nanowire, as predicted by the VLS model [14]. Nevertheless, it should be noticed that some HAADF images revealed the presence of some nanoparticles on the SiNS surface (see point C in the inset of Figure 4b), from which no SiNW emerges. EDX spectra were acquired at regions exhibiting no contrast on the SiNSs (point D) and on the dark spots present on the SiNS surface (point C). It was observed that, while the peaks of Au and C come from the supporting lacey carbon Au grid, only Si and O signals are found at point D, whereas point C indicates the additional presence of iron corresponding to the nanoparticle. In the literature, it has been demonstrated that Fe-catalyzed VLS growth cannot take place under temperatures below 1150 °C [13]. It should be considered that a temperature gradient is generated inside the ICP chamber and temperatures lower than 1150 °C can be reached at the bottom [16]. It is noteworthy to recall that the ICP process was designed for the synthesis of spherical Si particles. This leads us to suppose that, while the synthesis of spherical Si takes place, the self-assembly of SiNWs via VLS does not occur on those SiNS formed at temperatures lower than 1150 °C, i.e., at the bottom of the ICP machine [16]. Instead, SiNWs assembled via the VLS mechanism grown from SiNSs formed closer to the center of the ICP reactor, where the temperature is much higher.

## Conclusion

In conclusion, the combination of EFTEM, HAAD-STEM tomography and STEM-EDX spectroscopy was essential to confirm the growth mechanism of short SiNWs grown via the above-described ICP process. Indeed, by revealing the 3D structure of our ICP-produced Si nanostructures, it was possible to pinpoint the presence of small iron-containing nanoparticles at the top of the SiNWs. Moreover, EFTEM images revealed that the Si core of the SiNSs acted as a substrate from which the SiNWs grow. Metal-containing nanoparticles at the top of the SiNWs are an evident feature of VLS-grown SiNWs, where the growth is expected to start from the crystalline Si substrate. Hence, we conclude that the VLS mechanism is responsible for the growth of short SiNWs in the above-described ICP process, provided that a small amount of Fe is present during the spheroidization process. In this way, the ICP technique can be seen as a prospect for the synthesis of SiNWs via the VLS mechanism after suitable optimization of the process.

## Experimental

The inductively coupled plasma process [10] is conventionally exploited to transform a rough Si powder feedstock (irregular particles with mean size of 115 µm and 99.5% purity) into spherical microspheres of equivalent diameter. To this aim, the Si feedstock is introduced via an Ar carrier gas in the ICP reactor, where an Ar/H_2_ plasma is maintained at extremely high temperature (on the order of ≈10,000 K). The Si particles partially melt in-flight and, while being carried down in the first collector of the ICP machine, re-condensate as silicon spheres due to the surface-energy minimization principle. During the formation of the microspheres, a fraction of the Si feedstock sublimates and the molecules in the vapor phase aggregate in the form of lighter Si nanostructures. These are transported downstream in the reactor towards a second collector where they accumulate in the form of Si nanopowder. To perform our studies, this Si nanopowder was collected, dispersed into isopropyl alcohol, and sonicated for 5 min. By dropcasting this solution on Si substrates and on lacey carbon Cu or Au grids, both scanning electron microscope (SEM) imaging and TEM-based analyses were performed, respectively.

The SEM characterizations were carried out using a field emission gun (FEG) Zeiss Supra25. TEM analyses were performed with a JEOL JEM 2010F operating at 200 kV and with a spherical aberration probe corrected cold FEG ARM JEOL (0.27 eV energy spread) operating at 100 keV and equipped with a large area (100 mm^2^) EDX silicon drift detector with an energy resolution of 127 eV. The former was used to realise the EFTEM analysis on individual SiNWs, while the latter was used to accomplish the STEM-EDX analysis with a sub-nanometer probe and ET in STEM mode by using a HAADF detector. Electron tomography was conducted by using a single-tilt Fischione tomography sample holder (±60° range). Finally, the Composer Kai software was used for the reconstruction based on the filtered back projection (FBP) alghoritm, while the Visualiser Kai software was used for visualization.

## Supporting Information

File 1 A video showing the 3D reconstructed volume of a SiNS and VLS-grown SiNWs with an Fe nanoparticle on top. The image presented in Figure 3 was extracted from this video.
